# Phylogenetic and genomic diversity in isolates from the globally distributed *Acinetobacter baumannii* ST25 lineage

**DOI:** 10.1038/srep15188

**Published:** 2015-10-14

**Authors:** Jason W. Sahl, Mariateresa Del Franco, Spyros Pournaras, Rebecca E. Colman, Nabil Karah, Lenie Dijkshoorn, Raffaele Zarrilli

**Affiliations:** 1 Translational Genomics Research Institute, Flagstaff, AZ, USA; 2 Center for Microbial Genetics and Genomics, Northern Arizona University, Flagstaff, AZ, USA; 3 Department of Public Health, University of Naples “Federico II”, Naples, Italy; 4 Department of Microbiology, Medical School, University of Athens, Athens, Greece; 5 Department of Molecular Biology, Umeå University, Umeå, Sweden; 6 Department of Infectious Diseases, Leiden University Medical Centre, Leiden, The Netherlands

## Abstract

*Acinetobacter baumannii* is a globally distributed nosocomial pathogen that has gained interest due to its resistance to most currently used antimicrobials. Whole genome sequencing (WGS) and phylogenetics has begun to reveal the global genetic diversity of this pathogen. The evolution of *A. baumannii* has largely been defined by recombination, punctuated by the emergence and proliferation of defined clonal lineages. In this study we sequenced seven genomes from the sequence type (ST)25 lineage and compared them to 12 ST25 genomes deposited in public databases. A recombination analysis identified multiple genomic regions that are homoplasious in the ST25 phylogeny, indicating active or historical recombination. Genes associated with antimicrobial resistance were differentially distributed between ST25 genomes, which matched our laboratory-based antimicrobial susceptibility typing. Differences were also observed in biofilm formation between ST25 isolates, which were demonstrated to produce significantly more extensive biofilm than an isolate from the ST1 clonal lineage. These results demonstrate that within *A. baumannii*, even a fairly recently derived monophyletic lineage can still exhibit significant genotypic and phenotypic diversity. These results have implications for associating outbreaks with sequence typing as well as understanding mechanisms behind the global propagation of successful *A. baumannii* lineages.

*Acinetobacter baumannii* is an emergent nosocomial pathogen of increasing interest due to its widespread resistance to antimicrobials^1^. *A. baumannii* is truly a global pathogen, with isolates collected from hospitals around the world^2^,^3^, including injured soldiers from Iraq^4^ and Afghanistan^5^. The concern is the emergence of multidrug-resistant (MDR)^6^ and extremely drug-resistant (XDR)^7^ isolates that are resistant to most currently used therapeutics. Genes that confer resistance in *A. baumannii* have been documented, including class D beta-lactamases^8^, such as bla_OXA-51-like_, which appears to be highly conserved across *A. baumannii*^9^. The insertion element IS*Aba*1 is required for carbapenem resistance in bla_OXA-51-like_ positive isolates^10^.

The genome of *A. baumannii* is highly plastic^11^, with much of the evolution characterized by recombination^12^ and horizontal gene transfer^13^. The core genome phylogeny of *A. baumannii* demonstrates highly divergent genomes, with the emergence of a few highly successful clonal lineages^12^,^14^. While the evolution of these lineages is anticipated to be clonal, no in depth evolutionary studies have been performed to look at the fine scale evolution, recombination, and gene composition of these clades.

Infections caused by *A. baumannii* are increasing worldwide, possibly due to the rapid expansion of a selected number of genetically distinct lineages^12^,^14^. Three of these lineages, known as international clones I to III, represent globally distributed and ubiquitous clades^15^. Other successful lineages, which spread in single institutions and/or worldwide, have been identified in the population structure of *A. baumannii* using different genotyping methods, including sequence type ST25^14^. *A. baumannii* strains assigned to ST25 were responsible for epidemics in different European countries^16^,^17^,^18^,^19^,^20^ and the United Arab Emirates^21^ and were isolated as endemic or sporadic isolates in South America^22^ and Asia^18^, respectively. ST25 genomes are of increasing interest due to increasing antimicrobial resistance^14^ found within novel genomic resistance elements^23^.

The aim of the current study was to analyze the genomic epidemiology of 19 *A. baumannii* strains belonging to the ST25 lineage according to Pasteur’s MLST scheme^15^. Understanding the composition and evolution of one successful global lineage may help in understanding the genetic basis for the emergence and proliferation of global clones of *A. baumannii*.

## Methods

### Isolates

The collection of ST25 isolates analyzed in this study includes 19 strains: three sporadic strains from Leiden’s collection isolated during 1985, 2000 and 2002; 13 strains representative of epidemics or endemic circulation in different countries; three additional sporadic isolates selected because of their antimicrobial susceptibility profile and mechanisms of antimicrobial resistance^15^,^16^,^17^,^18^,^19^,^20^,^21^,^22^ (Table 1). Seven of these isolates were chosen for sequencing.

### Pulsed-field gel electrophoresis (PFGE) typing and dendrogram analysis

*ApaI* DNA macrorestriction and PFGE of *A. baumannii* isolates were performed as previously reported^24^. PFGE profiles were compared using the GelCompar II v. 4.6 software package (Applied Maths, Sint-Martens-Latem, Belgium). Clustering was based on the un-weighted pair-group method with arithmetic averages (UPGMA). The Dice correlation coefficient was used to analyze the similarities of the banding patterns with a tolerance of 1%. Interpretation of chromosomal DNA restriction patterns was based on the criteria of Tenover *et al.*^25^ and also on a similarity of >85% at dendrogram analysis, to indicate strain relatedness.

### MLST typing

Multi-locus sequence typing (MLST) analysis was performed using the Institut Pasteur’s MLST scheme as previously described^15^. Allele sequence and MLST profile definitions were assigned using the sequence and profile definitions available at http://pubmlst.org/abaumannii/. The MLST results were confirmed from the whole genome sequence analysis using a publically available script: https://github.com/Victorian-Bioinformatics-Consortium/mlst.

### DNA extraction, sequencing, assembly

DNA was extracted with the GenElute DNA extraction kit (Sigma-Aldrich, Milan, Italy). Sequence libraries were generated from extracted DNA as reported previously^9^. Genomes were sequenced to high depth on the IlluminaMiSeq platform. Resulting reads were adapter trimmed with Trimmomatic^26^, error corrected with Hammer^27^, and assembled with SPAdes v3.1^28^. The read coverage across each contig was evaluated, and contigs of an anomalous coverage, due to read crossover in multiplexed runs, were manually removed. The assembly stats for each genome are shown in Supplementary Table S1. All assemblies and raw reads were deposited in public databases (accession numbers in Supplementary Table S1). Annotation was performed with the NCBI PGAP pipeline.

### Antimicrobial susceptibility testing

Antimicrobial susceptibility testing was performed using the Vitek 2 system (bioMérieux, Marcy l’ Étoile, France). Imipenem, meropenem and colistin minimum inhibitory concentrations (MICs) were determined by agar dilution and Etest (bioMérieux) and interpreted using the EUCAST^29^ and CLSI 2012^30^ interpretative criteria.

### *in silico* antimicrobial susceptibility profiling

To identify previously characterized genes associated with antimicrobial resistance in our dataset, raw reads were mapped to the ResFinder database^31^ with the SRST2 pipeline^32^; raw reads were used to determine the percentage of the reference gene covered, but also could identify variants compared to the reference database. SRST2 produces a table of all positive hits identified in each genome.

### Biofilm formation

Biofilm formation was determined as previously described^55^. Three independent experiments, each one performed in triplicate, were conducted for each strain. Biofilms were grown in the presence and absence of 0.5 mg/L imipenem.

### Cell adhesion assays

Adherence of *A. baumannii* strains to A549 cells (human type 2 pneumocytes) was determined as described previously^55^, with minor modifications. In brief, ~10^5^ A549 cells were infected with ~10^7^ bacterial CFU and incubated for 60 min at 37 °C in 5% CO2 (v/v) atmosphere. Non-adherent bacterial cells were removed by washing with PBS. Infected cells were lysed by the addition of 1 ml distilled water and serial 10-fold dilutions were plated on LB agar to determine the number of CFU of adherent bacteria. To determine adherent and invading bacteria, A549 cells were infected with *A. baumannii* strains as described above. The monolayers were then treated with 1 ml of fresh culture medium containing 5 mg/L of colistin sulfate (Sigma-Aldrich, Milan, Italy) for 30 min, the shortest time point that resulted in the killing of all extracellular bacteria added to the monolayers. Afterwards, the cells were washed with PBS, harvested with trypsin, and lysed with sterile distilled water. Dilutions from harvested samples were inoculated on LB agar plates and bacterial colony counts were estimated after overnight incubation at 37 °C. Each experiment was performed in triplicate.

### Statistical analysis

Data were analyzed using a Statistical Package for the Social Sciences Version 13.0 (SPSS Inc., Chicago, IL, USA). Differences between mean values were tested for significance by performing either unpaired, two-tailed Student’s t-tests or one-way ANOVA analysis followed by Tukey’s multiple-comparison test, when appropriate. A P value < 0.05 was considered to be statistically significant. Correlations were evaluated by regression analysis using the Pearson’s correlation coefficient (r).

### Single nucleotide polymorphism (SNP) identification and phylogenetics

For ST25 comparisons, all SNPs were identified by mapping raw reads against *A. baumannii* AB307-0294 (NC_011595)^33^ with BWA-MEM^34^ and calling SNPs with the UnifiedGenotyper method in GATK^35^. For external genome assemblies, whole genome alignments were generated with nucmer^36^ and variants were identified by direct mapping of each query to the reference. These methods were wrapped by the Northern Arizona SNP Pipeline (NASP) (http://tgennorth.github.io/NASP/)^37^. A phylogeny was inferred from the resulting concatenated SNP alignment with a maximum likelihood algorithm in RaxML v8^38^. The Retention Index (RI) value^39^, which demonstrates how consistent the nucleotide character states are with the phylogeny, was calculated with Phangorn^40^.

For the global *A. baumannii* phylogeny, a set of 572 reference genomes (Supplementary Table S2) were downloaded from Patric^41^. Genome assemblies were aligned against AB307-0294 with NASP. A maximum likelihood phylogeny was inferred on this alignment with RaxML. Genomes were pruned from the phylogeny to only reflect the major sequence types. Clades were collapsed in ARB^42^.

### LS-BSR analysis

To look for differential gene conservation, the Large-Scale Blast Score Ratio (LS-BSR) pipeline^43^ was employed. In this method, all coding regions (CDSs) predicted by Prodigal^44^ are clustered with USEARCH^45^ at an ID of 90%. Each resulting centroid, which is the most representative sequence of each cluster, is then aligned against itself with BLAT^46^ to obtain the reference bit score. Each centroid is then separately aligned against each genome assembly with BLAT to obtain the query bit score. Dividing the query bit by the reference bit score returns the BLAST Score Ratio (BSR)^47^. Unique genomic regions were identified by comparing all CDSs between groups and considering a region to be unique if it had a BSR value > 0.8 in target genomes and a BSR value < 0.4 in all non-target genomes.

### Recombination analysis

The FASTA output of NASP was converted to NEXUS using Readseq^48^. The Retention Index for each base was then calculated with Paup v4a140^49^; the specific Paup commands are publicly available (https://gist.github.com/jasonsahl/a66afa55371d7d916a0e). The SNP density (SD), or number of parsimony-informative (PI) SNPs across a genomic interval, was calculated across 1-Kb, non-overlapping windows, compared to the reference genome of *A. baumannii* AB307-0294; PI SNPs are those that that contain at least two types of nucleotides and occur in a minimum of two genomes. The number of homoplasious SNPs, based on a per-base RI value < 0.5, was also calculated across the same window. The Homoplasy Density (HD) value was calculated by dividing the number of homoplasious SNPs (those SNPs that are inconsistent with the tree topology) by the total number of PI SNPs; a script to perform these functions has been published previously^50^. Visualization was performed by Circos^51^. Core genome regions, or those regions conserved across all genomes tested, were identified from the NASP output, where a call was made in all genomes.

### Plasmid analysis

CDSs predicted by Prodigal for 40 plasmids identified in *A. baumannii* (Supplementary Table S3) were mapped across ST25 genomes with LS-BSR and BLAT. Following manual curation of screened CDSs, the conservation of genes in associated plasmids was visualized with the interactive tree of life^52^. Only a subset of CDSs was selected to demonstrate the variability in plasmid content across isolates.

### Gene screen

The distribution of several genes associated with virulence or antimicrobial resistance was determined across all ST25 genomes. This included AbaR1, which has previously been shown to be missing from *A. baumannii* 4190^53^. This region (Coordinates 3702770-3602770 in *A. baumannii* AYE) was parsed out of *A. baumannii* AYE and coding regions were predicted with Prodigal. Other resistance islands, including AbaR4 (JN107991) and the G7 plasmid that contains AbaR3 (KF669606) were also screened. All CDSs were then compared against all ST25 genomes with LS-BSR and BLAT. An additional set of genes previously associated with virulence in *A. baumannii* (Supplementary Table S4) was also screened against ST25 genomes with LS-BSR.

## Results

### Isolates analyzed

**Figure 1:**
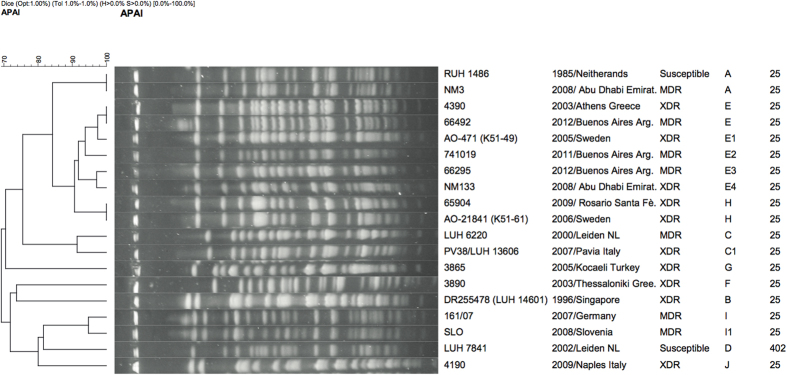
Genotypic analysis of PFGE profiles of *A. baumannii* strains included in the study. Percentage of similarity at dendogram analysis and position and tolerance values of the DICE correlation coefficient used in clustering are shown. Sizes in kilobases (kb) of lambda DNA molecular mass markers are indicated above the PFGE profiles. Strain number, year/country of isolation, PFGE types and subtypes and Multi-locus Sequence types are shown on the right of each profile.

In addition to ST25 genomes deposited in public databases, we selected a set of isolates to expand the diversity of this global lineage. The following seven isolates in our collection were selected for WGS analysis in addition to WGS data of strain 4190 already available in GenBank^54^: strains RUH1486 and NM3 are susceptible and MDR epidemic isolates, respectively; carbapenem-resistant strains 4390 and 741019 carry different carbapenemase genes (*bla*_OXA-58_ versus *bla*_OXA-23_) but both belong to the major PFGE type E (Fig. 1), isolated during epidemics in different countries (Table 1); strain 161/07 contains a distinct carbapenemase (NDM-1); strain LUH6220 has a MDR phenotype but is susceptible to carbapenems; strain LUH7841 is susceptible to most antimicrobials (Supplementary Table S5).

### PFGE Analysis

PFGE analysis identified ten unrelated PFGE types (A-J), with six PFGE subtypes within these types (Fig. 1) (C1, E1-E4, I1). Interestingly, PFGE type A included the susceptible first isolate of our collection (RUH1486) and the NM3 MDR strain isolated during an epidemic in United Arab Emirates; six strains from Greece, Argentina, Sweden or Abu Dhabi Emirates were assigned to PFGE type E or PFGE subtypes E1-E4, while two XDR isolates from Argentina and Sweden showed identical PFGE type H (Table 1 and Fig. 1).

### MLST

MLST with the Pasteur system^15^ was performed on 19 ST25 genomes, although only 7 were subjected to whole genome sequencing (WGS). Of all ST25 isolates analyzed, including a set of reference genomes from GenBank, eighteen were assigned to ST25, while one to ST402 (LUH7841), which is a single-locus variant of ST25. *In silico* MLST confirmed sequence type assignments for all genomes where WGS data exists (Supplementary Table S1).

### Antimicrobial susceptibility testing

The antimicrobial susceptibility profiles of 19 ST25 *A. baumannii* strains included in the study are shown in Supplementary Table S5. Two strains were classified as susceptible, six and eleven as MDR and XDR, respectively, according to Magiorakos *et al.*, 2012^29^. Five out of six MDR strains and all 11 XDR strains showed resistance to carbapenems and contained class D or class B carbapenemases.

### *In silico* antimicrobial resistance profiling

As a complement to the laboratory-determined antimicrobial susceptibility profiles, *in silico* profiles were generated for each sequenced genome, using the ResFinder database^31^ in conjunction with the SRST2 pipeline^32^. The results demonstrate that resistance mechanisms were identified in the genomes tested for only a few classes of antimicrobials (Table 2), demonstrating limitations in predicting the resistance phenotype from the genotype. Carbapenemase genes were found in the genomes of 161/07 (*bla*_NDM-1,_ *bla*_OXA-64_), 741019 and NM3 (*bla*_OXA-64,_ *bla*_OXA-23_), and 4390 and LUH6220 (*bla*_OXA-64_). The *bla*_OXA-64_ gene (AY750907), which is also known as *bla*_OXA-51-like_, is conserved in all ST25 genomes tested (Table 2). However, the IS*Aba*1 insertion sequence is missing in carbapenem-susceptible isolates that are *bla*_OXA-64_ positive, while present in resistant isolates, which confirms published results that this sequence is required for carbapenem resistance^10^.

### Biofilm formation and pneumocyte adherence

**Figure 2:**
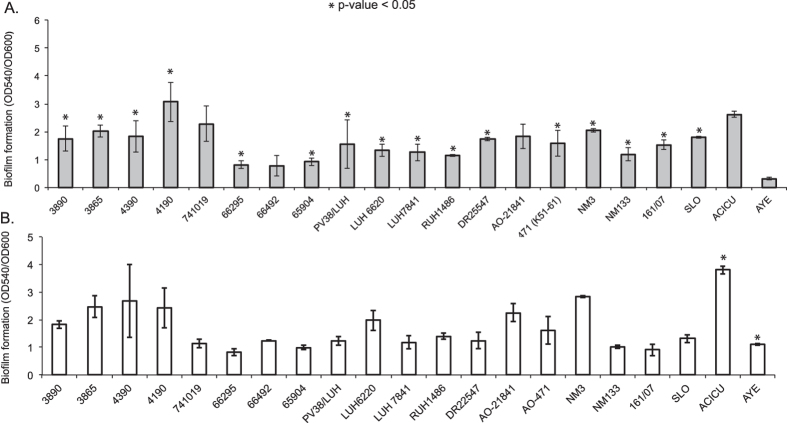
Biofilm variation between ST25 genomes, a ST1 genome (AYE), and a ST2 genome (ACICU). Error bars represent the standard deviation between biological replicates. Differences in biofilm production were calculated with a two-tailed t-test. Isolates were grown in the absence (**A**) or presence (**B**) of imipenem (0.5 mg/L).

Our previous results demonstrated that the ability to form biofilm and adherence to cultured pneumocytes was significantly higher for *A. baumannnii* strains assigned to ST25 and ST2 compared to other STs^55^. The biofilm growth on abiotic surfaces and adherence/invasion to cultured A549 pneumocytes were assessed for the 19 strains included in this study. As demonstrated in Fig. 2 panel A, the 19 strains assigned to ST25 and ST402 and strain ACICU assigned to ST2 generally demonstrated significantly (p < 0.05) greater biofilm growth than strain AYE assigned to ST1, although variability in biofilm growth was observed among ST25 strains. Exposure to sub-inhibitory concentrations of imipenem significantly (p < 0.05) stimulated biofilm growth in strains AYE and ACICU assigned to ST1 and ST2, respectively, but not in the strains assigned to ST25 or ST402 (Fig. 2, panel B). We next investigated the ability of *A. baumannii* strains to adhere to A549 human alveolar epithelial cells. All *A. baumannii* strains assigned to ST25 and ST2 (ACICU) strain showed a significantly higher adherence to A549 human bronchial cells compared with ST1 strain AYE (Supplementary Fig. S1) (p < 0.01). On the other hand, ST25 *A. baumannii* strains were not able to invade A549 cells human alveolar cells. Also, a similar number of bacteria adhered to A549 cells when the monolayers were incubated with *A. baumannii* strains for 60 min at 4 °C, i.e. under conditions that do not allow for tissue invasion.

### Sequencing and comparative genomics

**Figure 3:**
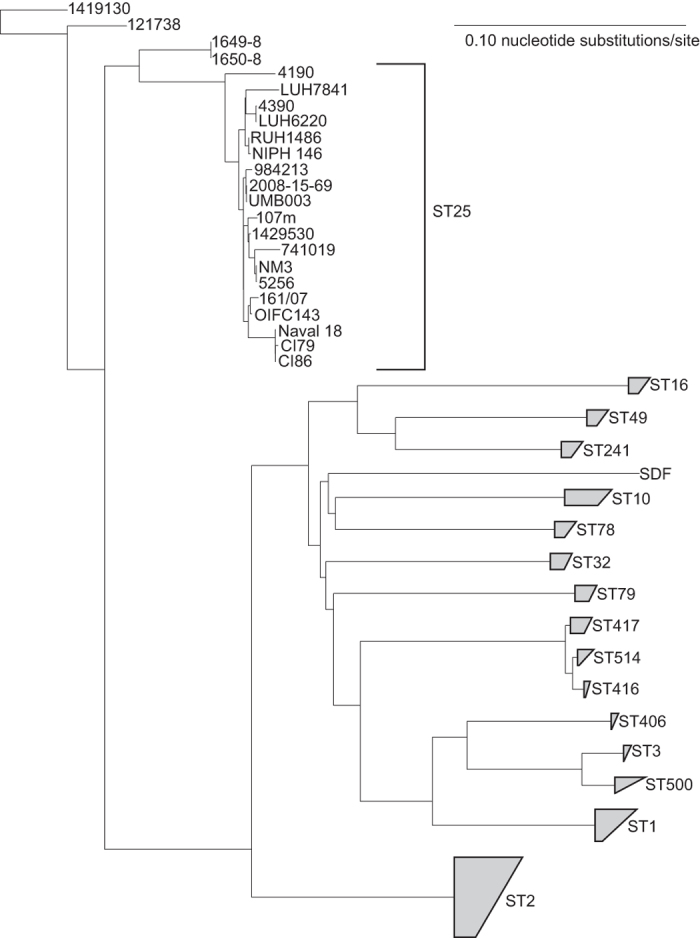
A core genome single nucleotide polymorphism (SNP) phylogeny of 597 *A. baumannii* genomes. The phylogeny was inferred with RAxML^38^ from a concatenation of ~104,000 SNPs compared to the reference genome of AB307-0294. Sequence types were identified from genome assemblies. Genomes without close relatives in established sequence types were manually pruned from the tree and groups were collapsed with ARB^42^. The phylogeny was rooted by first including an outgroup from *A. nosocomialis*, then re-running the analysis with only *A. baumannii* and rooting on the most basal genome from the original analysis.

**Figure 4:**
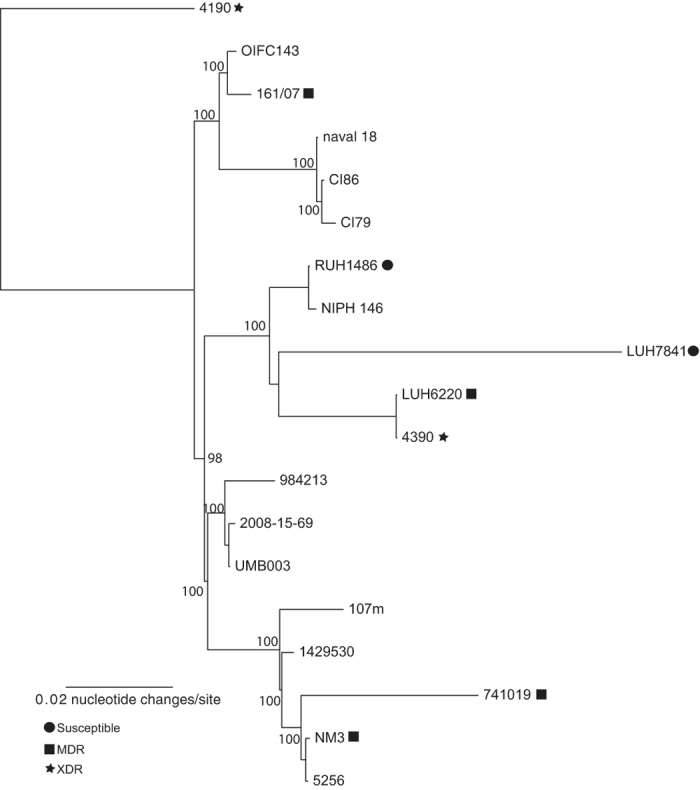
A core genome single nucleotide polymorphism (SNP) phylogeny of ST25 genomes. The phylogeny was inferred with RAxML from a concatenation of ~24,000 SNPs compared to the reference genome of AB307-0294^33^ with 100 bootstrap replicates. Each genome was annotated with its antimicrobial susceptibility information, where available. The tree was rooted according to the most basal genome isolated from the global phylogeny (Fig. 3).

WGS of seven strains was performed and compared to whole genome sequences of 12 *A. baumannii* strains assigned to ST25 available in GenBank (Supplementary Table S1) and to 572 non-ST25 *A. baumannii* reference genomes (Supplementary Table S2). The core genome phylogeny based on 1.15Mb of conserved sequence demonstrated the position of the ST25 lineage (Fig. 3) in relation to other globally-relevant lineages. The retention index (RI) of the concatenated SNP alignment was 0.85, demonstrating significant homoplasy likely due to recombination and introducing uncertainty in the phylogenetic placement, especially with regards to deeply branching nodes and long branches^56^. A phylogeny of just the ST25 genomes (Fig. 4) also demonstrated homoplasy (RI = 0.84), which demonstrates that although ST25 is a lineage with closely related genomes, the evolution of this group has also been partially driven by recombination; the core genome size of ST25 genomes in relation to AB307-0294 was 3Mb. As anticipated, the core genome phylogeny demonstrated much different relationships than were obtained by the PFGE cluster dendrogram (Figs 1 and 4). For example, genomes RUH1486 and NM3 both share the same PFGE type (Fig. 1), but are significantly different based on the core genome phylogeny (Fig. 4).

### Recombination in the ST25 lineage

**Figure 5:**
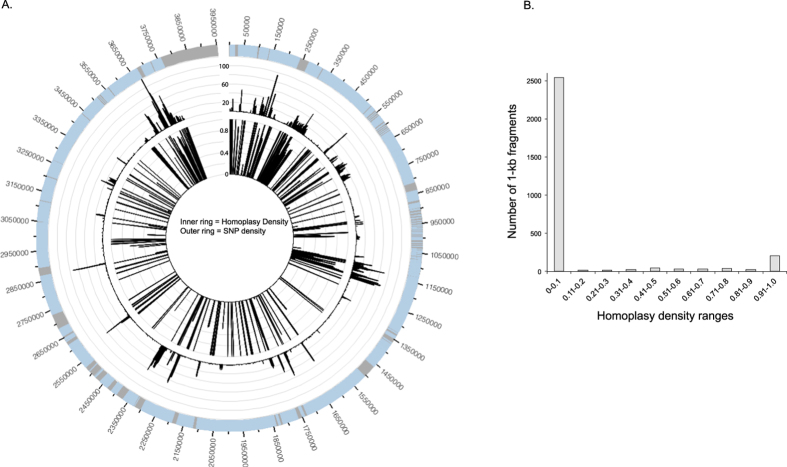
Homoplasy density (HD) ratio analysis of ST25 genomes. (Panel A): Parsimony informative (PI) single nucleotide polymorphisms (SNPs) were identified across 1-Kb, non-overlapping windows (SNP density, or SD), compared to the reference genome of AB307-0294. Homoplasious SNPs were identified by a Retention index value < 0.05. The HD was calculated by dividing the number of homoplasious SNPs by the total number of PI SNPs. The SD and HD values were visualized with Circos^51^. Core genome regions were identified where there was a call in all query genomes compared to the reference genome. (Panel B): The distribution of 1-kb, non-overlapping regions, based on the HD values.

To demonstrate both the extent and location of recombination in the ST25 lineage in relation to the genome of AB307-0294, a homoplasy density analysis was performed^50^. Considering all of the *A. baumannii* genomes (n = 597), the homoplasy appears to be distributed equally across the reference chromosome, with no isolated regions of recombination (Supplementary Fig. S2). When only considering the ST25 genomes, clear regions have likely been recombined between isolates (Fig. 5, panel A). However, fragments in the core genome, using AB307-0294 as the reference, still generally give a strong phylogenetic signal (Fig. 5, panel B). The annotation of selected regions associated with recombination is shown in Supplementary Table S6. A comprehensive list of HD values across all regions in the reference chromosome is also available (https://gist.github.com/5e1cab0b85c73de7c6d6.git).

### Plasmid composition

**Figure 6:**
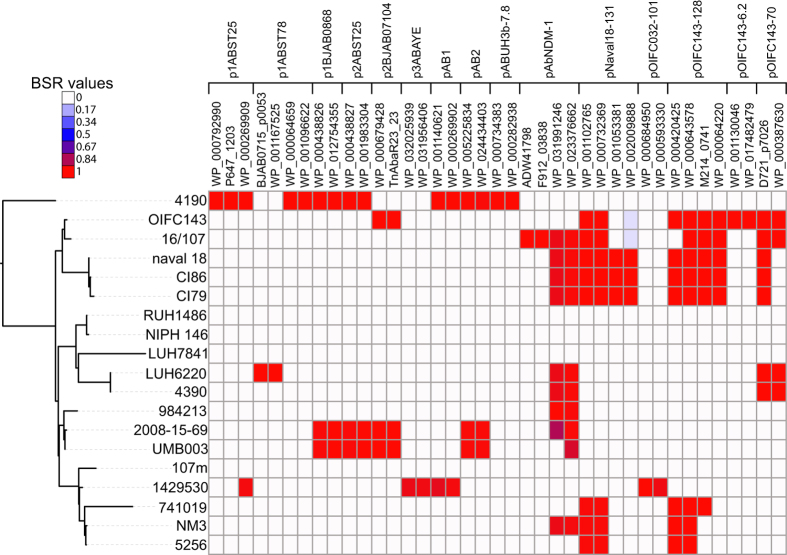
The ST25 phylogeny associated with a heatmap of coding regions predicted from plasmids identified in *A. baumannii* genomes. The heatmap was generated from LS-BSR^43^ output and was visualized with the interactive tree of life^52^. GenBank accession numbers are listed for each queried coding region.

The horizontal gene transfer of plasmids was analyzed in ST25 genomes, using the composition of 40 previously characterized *A. baumannii* plasmids (Supplementary Table S3). Coding regions were predicted for all the plasmids using Prodigal and they were compared against 19 ST25 genomes using LS-BSR. The results demonstrate that the plasmid content is highly variable across sequenced genomes (Fig. 6), although this method fails to discriminate between genes present on plasmids or the chromosome. The susceptible genome RUH1486 appeared to not contain any annotated plasmid.

### Unique genomic regions

The complete genetic content for 597 *A. baumannii* genomes was compared using LS-BSR. By using default values in LS-BSR, a single coding region was found to be present in ST25 genomes and absent from all others; this region corresponds to a large (~14600 nucleotides) hemagglutinin repeat protein (WP_002016208.1). While portions of this gene are conserved in other *A. baumannii* genomes, the complete gene structure is unique to ST25 genomes and could potentially serve as a diagnostic tool for the surveillance of this global lineage.

### Distribution of virulence associated genes

Virulence associated genes (Supplementary Table S4) were screened against ST25 genomes with LS-BSR and BLAT. The results demonstrate variability in composition across four genes. Perhaps the most striking difference is in *ompF*, which is highly conserved in only three genomes, each on the same branch of the phylogeny (Supplementary Fig. S3).

### Distribution of resistance islands

Coding regions from three previously characterized resistance islands were screened against ST25 genomes with LS-BSR. The results demonstrate that AbaR1 and AbaR4 were sparsely distributed across ST25 genomes (Supplementary Fig. S4). However, the AbaR3 resistance island was highly conserved across two lineages in the ST25 phylogeny, likely demonstrating independent acquisition.

### Discussion

*A. baumannii* is a globally distributed nosocomial pathogen associated with clinical infections that are difficult to treat due to widespread antimicrobial resistance. WGS has begun to demonstrate the phylogenetic diversity of this pathogen, which seems largely driven by homologous recombination^12^. And although phylogenetic diversity has been documented, many genomes sequenced to date fall into clearly defined clonal lineages, such as ST1 and ST2, which have been identified worldwide^14^. Genetic diversity within each of these sequence types has largely focused on the diversity of antimicrobial resistance islands^57^,^58^,^59^,^60^ or individual loci^61^. The focus of this study was to perform a comprehensive genomics analysis of the ST25 lineage, which was isolated in different countries and was responsible for epidemics worldwide^16^,^17^,^18^,^19^,^20^,^21^,^22^, to better understand the genotypic and phenotypic properties behind the worldwide distribution and evolution of a successful lineage of *A. baumannii*.

Phenotypic diversity was observed within ST25, including differences in biofilm formation, antimicrobial susceptibility, and pneumocyte adherence. In terms of biofilm formation, ST25 isolates produce a significantly higher amount of biofilm than a single ST1 representative in the absence of antimicrobials (Fig. 2, panel A). Although the relationship between biofilm formation and virulence has not been solidified in *A. baumannii*^62^, biofilms have been associated with resistance to antimicrobials^63^, pathogenensis^64^ as well as resistance to host factors^65^. The presence of imipenem did not affect biofilm production in ST25 isolates, suggesting that these mechanisms are constitutively expressed.

Antimicrobial susceptibility testing demonstrated varied susceptibility profiles within ST25 isolates (Supplementary Table S5). *In silico* profiles using WGS data against the ResFinder database could predict laboratory resistance for only a subset of antimicrobials (Table 2). This demonstrates that although antimicrobial resistance databases are useful for predicting resistance for some classes of antimicrobials, additional experimentation is required to fully understand the genetic basis for antimicrobial resistance in *A. baumannii*. In terms of adherence, ST25 genomes were demonstrated to adhere better than a representative from the ST1 clonal lineage. Although this may be due to the presence of a unique haemagglutinin identified in ST25 and absent from all other *A. baumannii* genomes, additional experimentation is required.

The genetic diversity of ST25 was demonstrated in multiple ways, including the visualization of a core genome single nucleotide polymorphism (SNP) phylogeny (Figs 3 and 4); a similar topology was observed compared to a recent CRISPR-subtyping analysis^66^. A homoplasy density approach demonstrated that much of the SNP density that defines the phylogenetic structure of ST25 is due to homoplasy (Fig. 5, panel A), most likely resulting from homologous recombination. The extent of homoplasy observed in the global phylogeny suggests that *A. baumannii* does not evolve in a tree like manner and different methods may better represent the evolution in this highly recombinant pathogen.

In addition to SNP analyses, comparative genomics demonstrated a much different gene content between ST25 genomes, primarily between mobile genetic elements. In the case of plasmids, the gene content was significantly different (Fig. 6), which was anticipated due to the movement of mobile genetic elements and has been demonstrated previously in *Acinetobacter*^67^. However, a screen of genes associated with virulence also demonstrated differences between closely related genomes (Supplementary Fig. S3). The variable distribution of genes across the ST25 dataset, including those associated with antimicrobial resistance islands (Supplementary Fig. S4), may help explain the variable phenotypes. Overall, these results demonstrate the problem with assuming that isolates have similar gene content or phenotypes based solely on MLST or PFGE type analyses. In terms of either assigning isolates to outbreaks or understanding the evolution of clonal lineages, WGS offers the resolution to untangle the relationships between seemingly related isolates.

## Additional Information

**How to cite this article**: Sahl, J. W. *et al.* Phylogenetic and genomic diversity in isolates from the globally distributed *Acinetobacter baumannii* ST25 lineage. *Sci. Rep.* **5**, 15188; doi: 10.1038/srep15188 (2015).

## Supplementary Material

Supplementary Information

## Figures and Tables

**Table 1:** Metadata associated with ST25 isolates analyzed in this study.

Isolate	Year/Country	Isolate Source	Pasteur ST^a^	Oxford ST^b^	Reference
RUH 1486	1985/Netherlands	Umbilicus	25	229	Diancourt *et al.*, 2010
NM3	2008/UAE	Sputum	25	229	Sonnevend *et al.* 2013
LUH 14601	1996/Singapore	Respiratory Tract	25	not typed	Unpublished
LUH 6220	2000/Netherlands	Sputum	25	not assigned	Unpublished
PV38/LUH 13606	2007/Italy	Upper Respiratory tract	25	not typed	Carretto *et al.* 2010
LUH 7841	2002/Netherlands	venuous catheter tip	402	229	Unpublished
4390	2003/Greece	Bronchial	25	not assigned	Gogou *et al.* 2011
66492	2012/Argentina	Blood	25	110	Stietz *et al.* 2013
AO-471	2005/Thailand	Wound	25	not typed	Karah *et al.* 2011
741019	2011/Argentina	Pleural fluid	25	not assigned	Stietz *et al.* 2013
66295	2012/Argentina	Blood	25	110	Stietz *et al.* 2013
NM133	2008/UAE	Bronchial aspirate	25	110	Sonnevend *et al.* 2013
3890	2003/Greece	Bronchial aspirate	25	not assigned	Di Popolo *et al.* 2011
3865	2005/Turkey	Blood	25	440	Di Popolo *et al.* 2011
AO-21841	2006/Sweden	Intra-abdominal isolate	25	not typed	Karah *et al.* 2011
65904	2009/Argentina	Inwelling catheter	25	not typed	Stietz *et al.* 2013
161/07	2007/Germany	Respiratory Tract	25	440	Bonnin *et al.* 2012
SLO	2008/Slovenia	Respiratory Tract	25	not typed	Bonnin *et al.* 2012
4190	2009/Italy	Blood	25	not assigned	Zarrilli *et al.* 2011

^a^ST assigned using Pasteur’sMLST scheme (Diancourt *et al.* 2010)

^b^ST assigned using Oxford’s MLST scheme (Bartual *et al.* 2005)

**Table 2:** *in silico* analysis of genes associated with antimicrobial resistance.

Genome	Resistance phenotype	Aminoglycosides	Beta-lactams	Sulfonamides	Tetracyclines
4390	XDR	X07753 (aphA6)	*bla*_OXA-64_	N/A	N/A
161/07	MDR	JN119852 (*aadB*), DQ336355 (OrfA), X00753 (aphA6), M96392 (*tnpA*)	*bla*_NDM-1_, *bla*_OXA-64_	GQ421466 *(glmM*)	N/A
741019	MDR	X57709 (aphA2), M96392 *(tnpA*)	*bla*_OXA-64_, *bla*_OXA-23_	GQ421466 (*glmM*)	AP000342 (*tetA*)
LUH7841	Susc.	N/A	*bla*_OXA-64_*	N/A	N/A
LUH6220	MDR	X57709 (aphA2), DQ336355 (OrfA), X07753 (aphA6)	*bla*_OXA-64_, *bla*_OXA-58_	N/A	N/A
NM3	MDR	X57709 (aphA2), M96392 *(tnpA*)	*bla*_OXA-64_, *bla*_OXA-23_	GQ421466 (*glmM*)	AP000342 (*tetA*)
RUH1486	Susc.	N/A	*bla*_OXA-64_*	N/A	N/A

N/A = no detection

^*^IS*Aba*1 negative
