# Supplementary material for: Phylogenetic and genomic diversity in isolates from the globally distributed Acinetobacter baumannii ST25 lineage
Source: Sci Rep. 2015 Oct 14;5:15188. doi: 10.1038/srep15188 (PMC4604477; doi:10.1038/srep15188)
Supplement: Supplementary Information [file srep15188-s1.pdf]

Supplementary Material

Title: Phylogenetic and genomic diversity in isolates from the globally distributed *Acinetobacter baumannii* ST25 lineage

Jason W. Sahl, Mariateresa Del Franco, Spyros Pournaras, Rebecca E. Colman, Nabil Karah, Lenie Dijkshoorn, Raffaele Zarrilli

Supplementary Table S1. Assembly stats and accession numbers for genomes sequenced in this study as well as reference genomes

Genome	Contigs	Assembly size	SRA accession	Assembly accession	Average depth of coverage	*in silico* MLST type
LUH7841	16	3801220	SRS853050	JZBX00000000	42x	402
LUH6220	63	3966581	SRS853048	JZBW00000000	52x	25
NM3	54	4100904	SRS853047	JZBV00000000	49x	25
741019	64	4055024	SRR1813762	JZBZ00000000	46x	25
161/07	63	4282986	SRS853053	JZCA00000000	46x	25
4390	157	3919530	SRS853051	JZBY00000000	42x	25
RUH1486	62	3888398	SRS853044	JZBU00000000	50x	25
5256	50	4158006	N/A	AHAI00000000	N/A	25
NIPH 146	1	3933401	N/A	NZ_KB849308.1	N/A	25
4190	398	4056516	N/A	AEPA00000000	N/A	25
UMB003	101	4140835	N/A	AEPM00000000	N/A	25
naval 18	55	4470438	N/A	AFDA00000000	N/A	25
OIFC143	8	4441327	N/A	AFDL00000000	N/A	25
107m	45	3880766	N/A	CBSG00000000	N/A	25
1429530	31	3985365	N/A	JEWM00000000	N/A	25
984213	449	4177035	N/A	JEVX00000000	N/A	25
CI79	85	4384434	N/A	AVOD00000000	N/A	25
CI86	89	4392843	N/A	AVOB00000000	N/A	25
2008-15-69	186	4255122	N/A	AMHN00000000	N/A	25

Supplementary Table S2. Accession information for reference genomes

Genome name	Accession
Acinetobacter_baumannii_1007214	JFWQ00000000
Acinetobacter_baumannii_1022959	JFDQ00000000
Acinetobacter_baumannii_1031433	JEYR00000000
Acinetobacter_baumannii_1032241	JEYS00000000
Acinetobacter_baumannii_1035119	JEVY00000000
Acinetobacter_baumannii_1036938	JEYT00000000
Acinetobacter_baumannii_1039621	JEYU00000000
Acinetobacter_baumannii_1040094	JEYV00000000
Acinetobacter_baumannii_1042969-1265	JEYW00000000
Acinetobacter_baumannii_1043794	JEYX00000000
Acinetobacter_baumannii_1043903	JEYY00000000
Acinetobacter_baumannii_1045092	JFFC00000000
Acinetobacter_baumannii_1046051	JEYZ00000000
Acinetobacter_baumannii_1046674	JEZA00000000
Acinetobacter_baumannii_1051176	JEZB00000000
Acinetobacter_baumannii_1051830	JEZC00000000
Acinetobacter_baumannii_10519	JFFF00000000
Acinetobacter_baumannii_1058283	JFWR00000000
Acinetobacter_baumannii_1062314	JFDR00000000
Acinetobacter_baumannii_1064293_45	JFDS00000000
Acinetobacter_baumannii_1075025	JEZD00000000
Acinetobacter_baumannii_107m	CBSG00000000
Acinetobacter_baumannii_1095464	JFDT00000000
Acinetobacter_baumannii_1096934	JEXM00000000
Acinetobacter_baumannii_1106579	JEXN00000000
Acinetobacter_baumannii_110912	JEVZ00000000
Acinetobacter_baumannii_11126	JFWS00000000
Acinetobacter_baumannii_1117819	JFWW00000000
Acinetobacter_baumannii_1121032	JEZE00000000
Acinetobacter_baumannii_1159076	JEWA00000000
Acinetobacter_baumannii_1170863	JFWT00000000
Acinetobacter_baumannii_1178044	JFDU00000000
Acinetobacter_baumannii_118362	JEWB00000000
Acinetobacter_baumannii_1188188	JFDV00000000
Acinetobacter_baumannii_1195185_11	JFEB00000000
Acinetobacter_baumannii_1202252	JEWC00000000
Acinetobacter_baumannii_1207552	JFEC00000000
Acinetobacter_baumannii_121738	JFDB00000000
Acinetobacter_baumannii_1232509	JFWY00000000
Acinetobacter_baumannii_1237202	JFWU00000000
Acinetobacter_baumannii_1237893	JFEA00000000
Acinetobacter_baumannii_1247182	JEZF00000000
Acinetobacter_baumannii_1262761-97	JFWV00000000
Acinetobacter_baumannii_1264936	JFED00000000
Acinetobacter_baumannii_1266220	JFXA00000000
Acinetobacter_baumannii_1267820	JEWD00000000
Acinetobacter_baumannii_1271213	JFDX00000000
Acinetobacter_baumannii_1271651	JFDZ00000000
Acinetobacter_baumannii_1276470-86	JFXE00000000
Acinetobacter_baumannii_1277411	JEXL00000000
Acinetobacter_baumannii_1287985	JFFG00000000
Acinetobacter_baumannii_1289546	JEZG00000000
Acinetobacter_baumannii_1291820	JEXK00000000
Acinetobacter_baumannii_1293320	JFEE00000000
Acinetobacter_baumannii_1294217	JEWF00000000
Acinetobacter_baumannii_1294222	JFXF00000000
Acinetobacter_baumannii_1295549	JFXB00000000
Acinetobacter_baumannii_1295743	JEWH00000000
Acinetobacter_baumannii_1296252	JFFU00000000
Acinetobacter_baumannii_1297549	JEVV00000000
Acinetobacter_baumannii_1297	JEZJ00000000
Acinetobacter_baumannii_136706	JEZH00000000
Acinetobacter_baumannii_1391434	JFXH00000000
Acinetobacter_baumannii_1392509	JFXJ00000000
Acinetobacter_baumannii_1397084	JEWJ00000000
Acinetobacter_baumannii_1397513	JFXI00000000
Acinetobacter_baumannii_1406182	JFXK00000000
Acinetobacter_baumannii_1406589	JFYI00000000
Acinetobacter_baumannii_1406750	JEWK00000000
Acinetobacter_baumannii_1412924	JFEF00000000
Acinetobacter_baumannii_1413735	JFWZ00000000
Acinetobacter_baumannii_1417041	JEYP00000000
Acinetobacter_baumannii_1419130	JEWL00000000
Acinetobacter_baumannii_14216	JFXN00000000
Acinetobacter_baumannii_1426993	JFCU00000000
Acinetobacter_baumannii_1428368	JFXM00000000
Acinetobacter_baumannii_1429530	JEWM00000000
Acinetobacter_baumannii_1440422	JEWO00000000
Acinetobacter_baumannii_1440750	JEXQ00000000
Acinetobacter_baumannii_144107	JEYE00000000
Acinetobacter_baumannii_145660	JFXU00000000
Acinetobacter_baumannii_1457504	JFEG00000000
Acinetobacter_baumannii_1461963	JEWQ00000000
Acinetobacter_baumannii_1462234	JEWR00000000
Acinetobacter_baumannii_1465485	JEWT00000000
Acinetobacter_baumannii_1471012	JFEH00000000
Acinetobacter_baumannii_1475764	JFEJ00000000
Acinetobacter_baumannii_1482820	JFEK00000000
Acinetobacter_baumannii_1488685	JFCT00000000
Acinetobacter_baumannii_1494580	JFXT00000000
Acinetobacter_baumannii_1525283	JEXR00000000
Acinetobacter_baumannii_1526966	JEXV00000000
Acinetobacter_baumannii_1533268	JFEI00000000
Acinetobacter_baumannii_1546444	JEXU00000000
Acinetobacter_baumannii_1552389	JFCS00000000
Acinetobacter_baumannii_1552818	JFCD00000000
Acinetobacter_baumannii_1552865	JEXY00000000
Acinetobacter_baumannii_1575710	JEYM00000000
Acinetobacter_baumannii_1593273	JEYL00000000
Acinetobacter_baumannii_1605	AUWL00000000
Acinetobacter_baumannii_1656-2	CP001921
Acinetobacter_baumannii_17534	JEYQ00000000
Acinetobacter_baumannii_213697	JFYE00000000
Acinetobacter_baumannii_214216	JFXR00000000
Acinetobacter_baumannii_232184	JEYI00000000
Acinetobacter_baumannii_23671	JFEN00000000
Acinetobacter_baumannii_24812_10	JFDJ00000000
Acinetobacter_baumannii_24812_4	JFDI00000000
Acinetobacter_baumannii_24812_6	JFDH00000000
Acinetobacter_baumannii_24845_10	JFWN00000000
Acinetobacter_baumannii_24845_1	JFWM00000000
Acinetobacter_baumannii_24845_3	JFWL00000000
Acinetobacter_baumannii_24845_4	JFWK00000000
Acinetobacter_baumannii_24845_5	JFWJ00000000
Acinetobacter_baumannii_24845_6	JFWI00000000
Acinetobacter_baumannii_24845_7	JFWH00000000
Acinetobacter_baumannii_24845_8	JFWG00000000
Acinetobacter_baumannii_24845_9	JFWF00000000
Acinetobacter_baumannii_24860_10	JFDG00000000
Acinetobacter_baumannii_24860_2	JFDF00000000
Acinetobacter_baumannii_24860_4	JFDE00000000
Acinetobacter_baumannii_24860_7	JFDD00000000
Acinetobacter_baumannii_24860_8	JFDC00000000
Acinetobacter_baumannii_24975_1	JGAI00000000
Acinetobacter_baumannii_24975_4	JGAH00000000
Acinetobacter_baumannii_24975_5	JGAG00000000
Acinetobacter_baumannii_24975_7	JGAF00000000
Acinetobacter_baumannii_24975_9	JGAE00000000
Acinetobacter_baumannii_25253_10	JFWE00000000
Acinetobacter_baumannii_25253_1	JFWD00000000
Acinetobacter_baumannii_25253_3	JFWC00000000
Acinetobacter_baumannii_25253_4	JFWB00000000
Acinetobacter_baumannii_25253_5	JFWA00000000
Acinetobacter_baumannii_25253_6	JFVZ00000000
Acinetobacter_baumannii_25253_7	JFVY00000000
Acinetobacter_baumannii_25253_8	JFVX00000000
Acinetobacter_baumannii_25307_1	JFWO00000000
Acinetobacter_baumannii_25307_2	JFVW00000000
Acinetobacter_baumannii_25307_3	JFVV00000000
Acinetobacter_baumannii_25307_4	JFVU00000000
Acinetobacter_baumannii_25307_5	JFVT00000000
Acinetobacter_baumannii_25307_6	JFVS00000000
Acinetobacter_baumannii_25307_8	JFVR00000000
Acinetobacter_baumannii_25561_10	JGBG00000000
Acinetobacter_baumannii_25561_1	JGAD00000000
Acinetobacter_baumannii_25561_2	JGAC00000000
Acinetobacter_baumannii_25561_7	JGAB00000000
Acinetobacter_baumannii_25681_2	JGCI00000000
Acinetobacter_baumannii_25681_3	JGCH00000000
Acinetobacter_baumannii_25681_5	JGCG00000000
Acinetobacter_baumannii_25681_6	JGCF00000000
Acinetobacter_baumannii_25681_8	JGCE00000000
Acinetobacter_baumannii_25681_9	JGCD00000000
Acinetobacter_baumannii_25691_10	JGAS00000000
Acinetobacter_baumannii_25691_1	JGAW00000000
Acinetobacter_baumannii_25691_2	JGAV00000000
Acinetobacter_baumannii_25691_6	JGAU00000000
Acinetobacter_baumannii_25691_9	JGAT00000000
Acinetobacter_baumannii_25750_6	JGCC00000000
Acinetobacter_baumannii_25750_7	JGCB00000000
Acinetobacter_baumannii_25750_8	JGCA00000000
Acinetobacter_baumannii_25766_10	JGAX00000000
Acinetobacter_baumannii_25766_1	JGBF00000000
Acinetobacter_baumannii_25766_2	JGBE00000000
Acinetobacter_baumannii_25766_3	JGBD00000000
Acinetobacter_baumannii_25766_4	JGBC00000000
Acinetobacter_baumannii_25766_5	JGBB00000000
Acinetobacter_baumannii_25766_6	JGBA00000000
Acinetobacter_baumannii_25766_7	JGAZ00000000
Acinetobacter_baumannii_25766_9	JGAY00000000
Acinetobacter_baumannii_25935_10	JGAJ00000000
Acinetobacter_baumannii_25935_1	JGAR00000000
Acinetobacter_baumannii_25935_2	JGAQ00000000
Acinetobacter_baumannii_25935_4	JGAP00000000
Acinetobacter_baumannii_25935_5	JGAO00000000
Acinetobacter_baumannii_25935_6	JGAN00000000
Acinetobacter_baumannii_25935_7	JGAM00000000
Acinetobacter_baumannii_25935_8	JGAL00000000
Acinetobacter_baumannii_25935_9	JGAK00000000
Acinetobacter_baumannii_268680	JEYN00000000
Acinetobacter_baumannii_273929	JFEO00000000
Acinetobacter_baumannii_277047	JFXS00000000
Acinetobacter_baumannii_2887	JFEP00000000
Acinetobacter_baumannii_29280	JEZI00000000
Acinetobacter_baumannii_299505	JEWY00000000
Acinetobacter_baumannii_303002	JFXW00000000
Acinetobacter_baumannii_318814	JFEQ00000000
Acinetobacter_baumannii_323408	JFCZ00000000
Acinetobacter_baumannii_3390	JFER00000000
Acinetobacter_baumannii_339786	JFXO00000000
Acinetobacter_baumannii_34001	JEZK00000000
Acinetobacter_baumannii_342950	JEWZ00000000
Acinetobacter_baumannii_34654	JEZL00000000
Acinetobacter_baumannii_3909	AEOZ00000000
Acinetobacter_baumannii_397971	JEYD00000000
Acinetobacter_baumannii_3990	AEOY00000000
Acinetobacter_baumannii_400834	JEXA00000000
Acinetobacter_baumannii_4190	AEPA00000000
Acinetobacter_baumannii_426863	JFYF00000000
Acinetobacter_baumannii_42887	JEYG00000000
Acinetobacter_baumannii_43926	JEXW00000000
Acinetobacter_baumannii_44327_1	JGBZ00000000
Acinetobacter_baumannii_44327_2	JGBY00000000
Acinetobacter_baumannii_44327_4	JGBX00000000
Acinetobacter_baumannii_44327_5	JGBW00000000
Acinetobacter_baumannii_44327_6	JGBH00000000
Acinetobacter_baumannii_44327_7	JGBV00000000
Acinetobacter_baumannii_44327_8	JGBU00000000
Acinetobacter_baumannii_44467_10	JGBT00000000
Acinetobacter_baumannii_44467_1	JGBS00000000
Acinetobacter_baumannii_44467_2	JGBR00000000
Acinetobacter_baumannii_44467_3	JGBQ00000000
Acinetobacter_baumannii_44467_4	JGBP00000000
Acinetobacter_baumannii_44467_5	JGBO00000000
Acinetobacter_baumannii_44467_6	JGBN00000000
Acinetobacter_baumannii_44467_7	JGBM00000000
Acinetobacter_baumannii_44467_8	JGBL00000000
Acinetobacter_baumannii_44467_9	JGBK00000000
Acinetobacter_baumannii_44857_10	JGBJ00000000
Acinetobacter_baumannii_44857_9	JGBI00000000
Acinetobacter_baumannii_44895_10	JFVI00000000
Acinetobacter_baumannii_44895_1	JFVH00000000
Acinetobacter_baumannii_44895_2	JFVG00000000
Acinetobacter_baumannii_44895_3	JFVF00000000
Acinetobacter_baumannii_44895_4	JFVE00000000
Acinetobacter_baumannii_44895_5	JFVD00000000
Acinetobacter_baumannii_44895_6	JFVC00000000
Acinetobacter_baumannii_44895_8	JFVB00000000
Acinetobacter_baumannii_44895_9	JFVA00000000
Acinetobacter_baumannii_45002_10	JFUZ00000000
Acinetobacter_baumannii_45002_3	JFUY00000000
Acinetobacter_baumannii_45002_4	JFUX00000000
Acinetobacter_baumannii_45002_5	JFUW00000000
Acinetobacter_baumannii_45002_6	JFUV00000000
Acinetobacter_baumannii_45002_7	JFUU00000000
Acinetobacter_baumannii_45002_8	JFUT00000000
Acinetobacter_baumannii_457946	JFET00000000
Acinetobacter_baumannii_458282	JFCY00000000
Acinetobacter_baumannii_466215	JFXP00000000
Acinetobacter_baumannii_466760	JEXB00000000
Acinetobacter_baumannii_470922	JFCX00000000
Acinetobacter_baumannii_472237-1196	JEZM00000000
Acinetobacter_baumannii_472237-120	JFCW00000000
Acinetobacter_baumannii_4749	JEXC00000000
Acinetobacter_baumannii_480175	JFEU00000000
Acinetobacter_baumannii_48055	AOSP00000000
Acinetobacter_baumannii_50595	JEXP00000000
Acinetobacter_baumannii_532279	JEYH00000000
Acinetobacter_baumannii_532413	JEZO00000000
Acinetobacter_baumannii_53264	ALPW00000000
Acinetobacter_baumannii_541915	JFXQ00000000
Acinetobacter_baumannii_554515	JEXT00000000
Acinetobacter_baumannii_562700	JEZP00000000
Acinetobacter_baumannii_564012	JEZQ00000000
Acinetobacter_baumannii_58452	JEZV00000000
Acinetobacter_baumannii_607805	JFES00000000
Acinetobacter_baumannii_625974	JEXD00000000
Acinetobacter_baumannii_628418	JFXX00000000
Acinetobacter_baumannii_647609	JEXE00000000
Acinetobacter_baumannii_653020	JEZU00000000
Acinetobacter_baumannii_655378	JFCE00000000
Acinetobacter_baumannii_655555	JFEW00000000
Acinetobacter_baumannii_662545-1347	JEZR00000000
Acinetobacter_baumannii_70136	JFYK00000000
Acinetobacter_baumannii_722310	JFCV00000000
Acinetobacter_baumannii_724909	JEXF00000000
Acinetobacter_baumannii_730795	JFYB00000000
Acinetobacter_baumannii_73736	JFFE00000000
Acinetobacter_baumannii_737393	JFEY00000000
Acinetobacter_baumannii_755829	JFXY00000000
Acinetobacter_baumannii_781407	JEZS00000000
Acinetobacter_baumannii_7893	JFDK00000000
Acinetobacter_baumannii_796380-2102	JEZT00000000
Acinetobacter_baumannii_825610	JFEZ00000000
Acinetobacter_baumannii_831240	JEYO00000000
Acinetobacter_baumannii_83444	JEYC00000000
Acinetobacter_baumannii_836190	JFFA00000000
Acinetobacter_baumannii_846928	JFFD00000000
Acinetobacter_baumannii_923118	JFFB00000000
Acinetobacter_baumannii_929679-2095	JEZW00000000
Acinetobacter_baumannii_942133	JEZX00000000
Acinetobacter_baumannii_942194	JFAC00000000
Acinetobacter_baumannii_947299	JEZY00000000
Acinetobacter_baumannii_951631	JEXI00000000
Acinetobacter_baumannii_959073	JEZZ00000000
Acinetobacter_baumannii_972082	JFAA00000000
Acinetobacter_baumannii_981176	JFYH00000000
Acinetobacter_baumannii_984213	JEVX00000000
Acinetobacter_baumannii_98826	JFYJ00000000
Acinetobacter_baumannii_99063	JEXJ00000000
Acinetobacter_baumannii_993520	JFAB00000000
Acinetobacter_baumannii_A118	AEOW00000000
Acinetobacter_baumannii_AA-014	AMGA00000000
Acinetobacter_baumannii_AB0057	NC_011586
Acinetobacter_baumannii_AB056	NZ_ADGZ00000000
Acinetobacter_baumannii_AB058	NZ_ADHA00000000
Acinetobacter_baumannii_AB059	NZ_ADHB00000000
Acinetobacter_baumannii_AB07	CP006963
Acinetobacter_baumannii_Ab11111	AKAQ00000000
Acinetobacter_baumannii_AB_1536-8	AMHA00000000
Acinetobacter_baumannii_AB_1582-8	AMHB00000000
Acinetobacter_baumannii_AB_1583-8	AMHC00000000
Acinetobacter_baumannii_AB_1594-8	AMHD00000000
Acinetobacter_baumannii_AB_1595-8	AMHE00000000
Acinetobacter_baumannii_AB_1649-8	AMHF00000000
Acinetobacter_baumannii_AB_1650-8	AMHG00000000
Acinetobacter_baumannii_AB_1766_8	AMJO00000000
Acinetobacter_baumannii_AB1H8	ANNC00000000
Acinetobacter_baumannii_AB_2007-09-110-01	AMHH00000000
Acinetobacter_baumannii_AB_2007-16-25-01-1	AMHI00000000
Acinetobacter_baumannii_AB_2007-16-27-01	AMHJ00000000
Acinetobacter_baumannii_AB_2008-15-34-7	AMHK00000000
Acinetobacter_baumannii_AB_2008-15-45	AMHL00000000
Acinetobacter_baumannii_AB_2008-15-52	AMHM00000000
Acinetobacter_baumannii_AB_2008-15-69	AMHN00000000
Acinetobacter_baumannii_AB_2008-15-70	AMHO00000000
Acinetobacter_baumannii_AB_2008-15-71	AMHP00000000
Acinetobacter_baumannii_AB_2008-23-01-01-1	AMHQ00000000
Acinetobacter_baumannii_AB_2008-23-07-01-1	AMHR00000000
Acinetobacter_baumannii_AB_2009-04-01-7	AMHS00000000
Acinetobacter_baumannii_AB_2009-04-02-7	AMHT00000000
Acinetobacter_baumannii_AB210	AEOX00000000
Acinetobacter_baumannii_AB307-0294	NC_011595
Acinetobacter_baumannii_Ab33333	AKAS00000000
Acinetobacter_baumannii_AB405E4	ANND00000000
Acinetobacter_baumannii_Ab44444	AKAT00000000
Acinetobacter_baumannii_AB4857	AHAG00000000
Acinetobacter_baumannii_AB4A3	AOLU00000000
Acinetobacter_baumannii_AB5075	AHAH00000000
Acinetobacter_baumannii_AB_515-8	AMHU00000000
Acinetobacter_baumannii_AB5256	AHAI00000000
Acinetobacter_baumannii_AB5711	AHAJ00000000
Acinetobacter_baumannii_AB900	NZ_ABXK00000000
Acinetobacter_baumannii_AB_908-12	AMHV00000000
Acinetobacter_baumannii_AB_908-13	AMHW00000000
Acinetobacter_baumannii_AB_908-14-7	AMHX00000000
Acinetobacter_baumannii_AB_909-01-7	AMHY00000000
Acinetobacter_baumannii_AB_909-02-7	AMHZ00000000
Acinetobacter_baumannii_AB_909-05	AMIA00000000
Acinetobacter_baumannii_AB_909-14-7	AMIB00000000
Acinetobacter_baumannii_AB-HKU3-08	ATHY00000000
Acinetobacter_baumannii_AB-HKU3-10	ATHZ00000000
Acinetobacter_baumannii_ABIsac_ColiR	CAKB00000000
Acinetobacter_baumannii_ABIsac_ColiS	CAKA00000000
Acinetobacter_baumannii_ABNIH10	APAZ00000000
Acinetobacter_baumannii_ABNIH11	APBA00000000
Acinetobacter_baumannii_ABNIH13	APBB00000000
Acinetobacter_baumannii_ABNIH14	APBC00000000
Acinetobacter_baumannii_ABNIH15	APBD00000000
Acinetobacter_baumannii_ABNIH16	APBE00000000
Acinetobacter_baumannii_ABNIH17	APBF00000000
Acinetobacter_baumannii_ABNIH18	APBG00000000
Acinetobacter_baumannii_ABNIH19	APBH00000000
Acinetobacter_baumannii_ABNIH1	AFSZ00000000
Acinetobacter_baumannii_ABNIH20	APBI00000000
Acinetobacter_baumannii_ABNIH22	APBJ00000000
Acinetobacter_baumannii_ABNIH23	APBK00000000
Acinetobacter_baumannii_ABNIH24	APBL00000000
Acinetobacter_baumannii_ABNIH25	APBM00000000
Acinetobacter_baumannii_ABNIH26	AOGD00000000
Acinetobacter_baumannii_ABNIH2	AFTA00000000
Acinetobacter_baumannii_ABNIH3	AFTB00000000
Acinetobacter_baumannii_ABNIH4	AFTC00000000
Acinetobacter_baumannii_ABNIH5	APAW00000000
Acinetobacter_baumannii_ABNIH6	APAX00000000
Acinetobacter_baumannii_ABNIH7	APAY00000000
Acinetobacter_baumannii_AB_TG19617	AMID00000000
Acinetobacter_baumannii_AB_TG2018	AMIE00000000
Acinetobacter_baumannii_AB_TG2022	AMIF00000000
Acinetobacter_baumannii_AB_TG2023	AMIG00000000
Acinetobacter_baumannii_AB_TG2026	AMIH00000000
Acinetobacter_baumannii_AB_TG2028	AMII00000000
Acinetobacter_baumannii_AB_TG2030	AMIJ00000000
Acinetobacter_baumannii_AB_TG2031	AMIK00000000
Acinetobacter_baumannii_AB_TG2032	AMIL00000000
Acinetobacter_baumannii_AB_TG2631	AMIM00000000
Acinetobacter_baumannii_AB_TG27323	AMIN00000000
Acinetobacter_baumannii_AB_TG27327	AMIO00000000
Acinetobacter_baumannii_AB_TG27331	AMIP00000000
Acinetobacter_baumannii_AB_TG27335	AMIQ00000000
Acinetobacter_baumannii_AB_TG27339	AMIR00000000
Acinetobacter_baumannii_AB_TG27343	AMIS00000000
Acinetobacter_baumannii_AB_TG5064	AMIU00000000
Acinetobacter_baumannii_AC12	ALAM00000000
Acinetobacter_baumannii_AC30	ALXD00000000
Acinetobacter_baumannii_ACICU	NC_010611
Acinetobacter_baumannii_ANC_4097	APRF00000000
Acinetobacter_baumannii_ATCC_17978	NC_009085
Acinetobacter_baumannii_ATCC_19606	NZ_GG704572
Acinetobacter_baumannii_AYE	NC_010410
Acinetobacter_baumannii_BJAB07104	CP003846
Acinetobacter_baumannii_BJAB0715	CP003847
Acinetobacter_baumannii_BJAB0868	CP003849
Acinetobacter_baumannii_BZICU-2	ALOH00000000
Acinetobacter_baumannii_Canada_BC1	AMSZ00000000
Acinetobacter_baumannii_Canada_BC-5	AFDN00000000
Acinetobacter_baumannii_CI77	AVOC00000000
Acinetobacter_baumannii_CI79	AVOD00000000
Acinetobacter_baumannii_CI86	AVOB00000000
Acinetobacter_baumannii_D1279779	AERZ00000000
Acinetobacter_baumannii_DU202	AVGF00000000
Acinetobacter_baumannii_EGD-HP18	AVST00000000
Acinetobacter_baumannii_IS-116	AMGF00000000
Acinetobacter_baumannii_IS-123	ALII00000000
Acinetobacter_baumannii_IS-143	AMGE00000000
Acinetobacter_baumannii_IS-235	AMEI00000000
Acinetobacter_baumannii_IS-251	AMEJ00000000
Acinetobacter_baumannii_IS-58	AMGH00000000
Acinetobacter_baumannii_MDR_MMC4	AZNQ00000000
Acinetobacter_baumannii_MDR-TJ	AEOE00000000
Acinetobacter_baumannii_MDR-ZJ06	CP001937
Acinetobacter_baumannii_MRSN_57	JABT00000000
Acinetobacter_baumannii_MRSN_58	JABU00000000
Acinetobacter_baumannii_MRY09-0642	BASA00000000
Acinetobacter_baumannii_MRY10-0558	BASB00000000
Acinetobacter_baumannii_MRY12-0277	BASC00000000
Acinetobacter_baumannii_MSP4-16	AODW00000000
Acinetobacter_baumannii_Naval-113	AMZU00000000
Acinetobacter_baumannii_Naval-13	AMDR00000000
Acinetobacter_baumannii_Naval-17	AFDO00000000
Acinetobacter_baumannii_Naval-18	AFDA00000000
Acinetobacter_baumannii_Naval-21	AMSY00000000
Acinetobacter_baumannii_Naval-2	AMSX00000000
Acinetobacter_baumannii_Naval-57	AMFP00000000
Acinetobacter_baumannii_Naval-72	AMFI00000000
Acinetobacter_baumannii_Naval-78	AMFZ00000000
Acinetobacter_baumannii_Naval-81	AFDB00000000
Acinetobacter_baumannii_Naval-82	AMSW00000000
Acinetobacter_baumannii_Naval-83	AMFK00000000
Acinetobacter_baumannii_NIPH_1362	APOR00000000
Acinetobacter_baumannii_NIPH_146	APOU00000000
Acinetobacter_baumannii_NIPH_1669	APOQ00000000
Acinetobacter_baumannii_NIPH_1734	APOX00000000
Acinetobacter_baumannii_NIPH_190	APPL00000000
Acinetobacter_baumannii_NIPH_201	APQV00000000
Acinetobacter_baumannii_NIPH_2061	APOW00000000
Acinetobacter_baumannii_NIPH_24	APOF00000000
Acinetobacter_baumannii_NIPH_290	APRD00000000
Acinetobacter_baumannii_NIPH_329	APQY00000000
Acinetobacter_baumannii_NIPH_335	APQX00000000
Acinetobacter_baumannii_NIPH_410	ATGJ00000000
Acinetobacter_baumannii_NIPH_527	APQW00000000
Acinetobacter_baumannii_NIPH_528	APRB00000000
Acinetobacter_baumannii_NIPH_601	APQZ00000000
Acinetobacter_baumannii_NIPH_60	APPM00000000
Acinetobacter_baumannii_NIPH_615	APOV00000000
Acinetobacter_baumannii_NIPH_67	APRA00000000
Acinetobacter_baumannii_NIPH_70	APRC00000000
Acinetobacter_baumannii_NIPH_80	APRE00000000
Acinetobacter_baumannii_OIFC0162	AMFH00000000
Acinetobacter_baumannii_OIFC032	AFCZ00000000
Acinetobacter_baumannii_OIFC035	AMTB00000000
Acinetobacter_baumannii_OIFC047	AMFW00000000
Acinetobacter_baumannii_OIFC065	AMFV00000000
Acinetobacter_baumannii_OIFC074	AMDE00000000
Acinetobacter_baumannii_OIFC087	AMFS00000000
Acinetobacter_baumannii_OIFC098	AMDF00000000
Acinetobacter_baumannii_OIFC099	AMFT00000000
Acinetobacter_baumannii_OIFC109	ALAL00000000
Acinetobacter_baumannii_OIFC110	AMFL00000000
Acinetobacter_baumannii_OIFC111	AMFY00000000
Acinetobacter_baumannii_OIFC137	AFDK00000000
Acinetobacter_baumannii_OIFC143	AFDL00000000
Acinetobacter_baumannii_OIFC180	AMDQ00000000
Acinetobacter_baumannii_OIFC189	AFDM00000000
Acinetobacter_baumannii_OIFC338	AMFX00000000
Acinetobacter_baumannii_PR07	APWV00000000
Acinetobacter_baumannii_SDF	NC_010400
Acinetobacter_baumannii_str_Perm	AUZL00000000
Acinetobacter_baumannii_TCDC-AB0715	CP002522
Acinetobacter_baumannii_TG00314	ASER00000000
Acinetobacter_baumannii_TG02011	ASES00000000
Acinetobacter_baumannii_TG02017	ASET00000000
Acinetobacter_baumannii_TG07725	ASEU00000000
Acinetobacter_baumannii_TG15233	ASEV00000000
Acinetobacter_baumannii_TG15234	ASEW00000000
Acinetobacter_baumannii_TG15236	ASEX00000000
Acinetobacter_baumannii_TG15237	ASEY00000000
Acinetobacter_baumannii_TG15238	ASEZ00000000
Acinetobacter_baumannii_TG15239	ASFA00000000
Acinetobacter_baumannii_TG15240	ASFB00000000
Acinetobacter_baumannii_TG15241	ASFC00000000
Acinetobacter_baumannii_TG15242	ASFD00000000
Acinetobacter_baumannii_TG19582	AMIV00000000
Acinetobacter_baumannii_TG2012	ASFE00000000
Acinetobacter_baumannii_TG2013	ASFF00000000
Acinetobacter_baumannii_TG2014	ASFG00000000
Acinetobacter_baumannii_TG20277	ASFH00000000
Acinetobacter_baumannii_TG20546	ASFI00000000
Acinetobacter_baumannii_TG22110	ASFJ00000000
Acinetobacter_baumannii_TG22112	ASFK00000000
Acinetobacter_baumannii_TG22142	ASFL00000000
Acinetobacter_baumannii_TG22146	ASFM00000000
Acinetobacter_baumannii_TG22148	ASFN00000000
Acinetobacter_baumannii_TG22150	ASFO00000000
Acinetobacter_baumannii_TG22190	ASFP00000000
Acinetobacter_baumannii_TG22192	ASFQ00000000
Acinetobacter_baumannii_TG22194	ASFR00000000
Acinetobacter_baumannii_TG22196	ASFS00000000
Acinetobacter_baumannii_TG22198	ASFT00000000
Acinetobacter_baumannii_TG22202	ASFU00000000
Acinetobacter_baumannii_TG22204	ASFV00000000
Acinetobacter_baumannii_TG22212	ASFW00000000
Acinetobacter_baumannii_TG22214	ASFX00000000
Acinetobacter_baumannii_TG22332	ASFY00000000
Acinetobacter_baumannii_TG22336	ASFZ00000000
Acinetobacter_baumannii_TG27295	ASGA00000000
Acinetobacter_baumannii_TG27299	ASGB00000000
Acinetobacter_baumannii_TG27307	ASGC00000000
Acinetobacter_baumannii_TG27311	ASGD00000000
Acinetobacter_baumannii_TG27315	ASGE00000000
Acinetobacter_baumannii_TG27319	ASGF00000000
Acinetobacter_baumannii_TG27371	ASGG00000000
Acinetobacter_baumannii_TG27379	ASGH00000000
Acinetobacter_baumannii_TG27383	ASGI00000000
Acinetobacter_baumannii_TG27387	ASGJ00000000
Acinetobacter_baumannii_TG27391	ASGK00000000

Supplementary Table S3. Accession information for plasmids screened in the current study

Plasmid name	Accession
p1ABAYE	NC_010401
p1ABSDF	NC_010395
p1ABST25	NZ_AEPA01000395
p1ABST78	NZ_AEOZ01000236
p1BJAB07104	NC_021727
p1BJAB0868	NC_021730
p2ABAYE	NC_010402
p2ABSDF	NC_010396
p2ABST25	NZ_AEPA01000396
p2BJAB07104	NC_021728
p2BJAB0868	NC_021731
p3ABAYE	NC_010404
p3ABSDF	NC_010398
p3BJAB0868	NC_021732
p4ABAYE	NC_010403
pAB0057	NC_011585
pAB1	NC_009083
pAB2	NC_009084
pABUH2a-5.6	AYFZ01000080
pABUH2b-5.4	AYFH01000057
pABUH3a-8.2	AYFH01000048
pABUH3b-7.8	AYFZ01000083
pAbNDM-1	NC_019985
pBJAB0715	NC_021734
pCanadaBC5-8.7	NZ_AFDN01000003
pD1279779	NC_020525
pMDR-ZJ06	NC_017172
pNaval18-131	NZ_AFDA02000009
pNaval18-5.7	NZ_AFDA02000010
pNaval18-6.1	NZ_AFDA02000006
pNaval18-7.0	NZ_AFDA02000011
pNaval18-74	NZ_AFDA02000007
pNaval18-8.4	NZ_AFDA02000008
pOIFC032-101	NZ_AFCZ02000003
pOIFC032-8.6	NZ_AFCZ02000004
pOIFC143-128	NZ_AFDL01000008
pOIFC143-2.3	NZ_AFDL01000005
pOIFC143-6.2	NZ_AFDL01000007
pOIFC143-70	NZ_AFDL01000006
pZW85p2	NC_023031

Supplementary Table S4. Virulence associated genes screened across ST25 genomes in this study

gene	annotation	accession	Publication (PMID)
zur	ferric uptake regulator family protein	A1S_0145	23236280
hcp	type VI secretion system effector, Hcp1 family	A1S_1296	23365692
bap	biofilm associated protein	ABAYE0792	22083703
entA	2,3-dihydroxybenzoate-2,3-dehydrogenase	A1S_2579	22570720
pmt	transporter-like protein gene	HM595762	22518144
csuE	protein CsuE	AAP43040	14663080
entE	enterobactin synthase subunit E	ABAYE1096	20853905
sc1	siderophore biosynthesis protein	ABAYE2004	18350144
bfmR	two-component regulatory system response regulator	YP_045456	18957593
envZ	Osmolarity sensor protein EnvZ	ABBFA_000285	22911967
epsA	EPS I polysaccharide export outer membrane protein	ABBFA_003459	22911967
ompF	Outer membrane porin F precursor	ABBFA_000621	22911967
ostA	Organic solvent tolerance protein OstA	ABBFA_001929	22911967
pbpG	Penicillin-binding protein 7/8	ABBFA_003295	22911967
ptk	Tyrosine-protein kinase Ptk	ABBFA_003461	22911967
rstA	Transcriptional regulatory protein RstA	ABBFA_002866	22911967
spsC	Perosamine synthase	ABBFA_003451	22911967

Supplementary Table S5. Antibiotic susceptibility profiles of *A. baumannii* strains included in this study.

Strain	MIC (µg/ml) of antibiotic														
	IPM	MER	COL	RIF	SAM	SXT	TIGEC	TETRA	CAZ	TZP	FEP	LEVOFL	CIP	GEN	TOB
3890	16	6	0.75	8	8	0.75	0.19	6	6	96	6	0.38	1	>256	64
4390	32	8	0.75	4	16	>32	0.125	8	192	>256	32	12	>32	64	16
66492	>32	>32	0.75	6	128	>32	0.38	24	>256	>256	>256	>32	>32	192	128
AO-471 (K51-49)	>32	>32	0.5	3	48	>32	0.25	4	>256	>256	128	8	>32	>256	128
741019	>32	>32	0.75	1.5	32	1	0.38	4	>256	>256	128	2	16	>256	>256
66295	>32	>32	0.75	>32	48	0.5	0.19	4	>256	>256	128	24	>32	1	1
NM133	>32	>32	0.75	>32	>256	>32	0.38	12	>256	>256	>256	6	>32	>256	>256
65904	>32	>32	0.75	4	64	>32	0.19	6	>256	>256	128	12	>32	192	64
AO-21841 (K51-61)	>32	>32	0.38	2	>256	>32	0.38	>256	>256	>256	>256	>32	>32	>256	128
RUH 1486	0.25	0.5	0.5	3	4	0.125	0.125	4	4	12	4	0.094	0.5	1	1
NM3	>32	>32	0.75	3	24	16	0.25	4	8	>256	48	4	>32	>256	192
LUH 6220	>32	32	0.5	8	0.5	0.5	0.5	32	8	>256	24	8	16	>256	64
PV38/LUH 13606	>32	>32	0.75	12	16	1	0.25	12	12	>256	16	3	8	2	2
3865	>32	>32	0.75	>32	>256	32	0.25	6	192	>256	256	12	>32	>256	128
SLO	>32	>32	0.5	3	>256	12	0.25	8	>256	>256	>256	16	>32	>256	128
161/07	>32	>32	0.5	3	>256	16	0.38	6	>256	>256	>256	12	>32	>256	128
LUH 7841	0.19	0.5	0.5	6	3	0.75	0.094	2	2	2	1.5	0.19	1	1.5	1
4190	>32	>32	0.75	4	24	0.094	0.38	32	>256	>256	192	8	>32	3	2
DR25547 (LUH 14601)	>32	>32	1	2	48	0.19	0.5	8	>256	>256	128	8	>32	>256	>256

Abbreviations: MIC, Minimum inhibitory concentration;IPM, imipenem; MER, meropenem; COL, colistin; RIF, rifampin; SAM, sulbactam-ampicillin; SXT, trimetoprim/sulfametoxazolo; TIGEC, tigecycline; TETRA, tetracycline; CAZ, ceftazidime ; TZP ,piperacillin/tazobactam ; FEP, cefepime ; LEVOFL, levofloxacin; CIP, ciprofloxacin; GEN, gentamicin ; TOB, tobramycin.

Supplementary Table S6. Regions in the reference chromosome associated with high SNP density in ST25 genomes

Chromosome	start coord	end coord	# Parsimony-informative SNPs	#Homoplasious SNPs	Homoplasy density ratio	locus tag(s)	Annotation
NC_011595	15001	16001	27	27	1.00	ABBFA_000013	Anhydro-N-acetylmuramic acid kinase(AnhMurNAc kinase)
NC_011595	196001	197001	53	45	0.85	ABBFA_000174,ABBFA_000175	transcriptional regulator lrpB,Alkanesulfonate monooxygenase
NC_011595	533001	534001	25	25	1.00	ABBFA_000509,ABBFA_000510	Acyl-CoA dehydrogenase, Enoyl-CoA hydratase,Enoyl-CoA hydratase
NC_011595	534001	535001	67	67	1.00	ABBFA_000510, ABBFA_000511	Enoyl-CoA hydratase, acetyl-CoA carboxylase
NC_011595	535001	536001	53	53	1.00	ABBFA_000511	acetyl-CoA carboxylase
NC_011595	627001	628001	21	21	1.00	ABBFA_000597,ABBFA_000598	Metallo-beta-lactamase, AhpC/TSA family protein
NC_011595	1176001	1177001	60	53	0.88	ABBFA_001079, ABBFA_001080	xanthine dehydrogenase, accessory protein XdhC
NC_011595	2280001	2281001	21	21	1.00	ABBFA_002139, ABBFA_002140	Major Facilitator Superfamily protein, enoyl-(acyl-carrier-protein) reductase

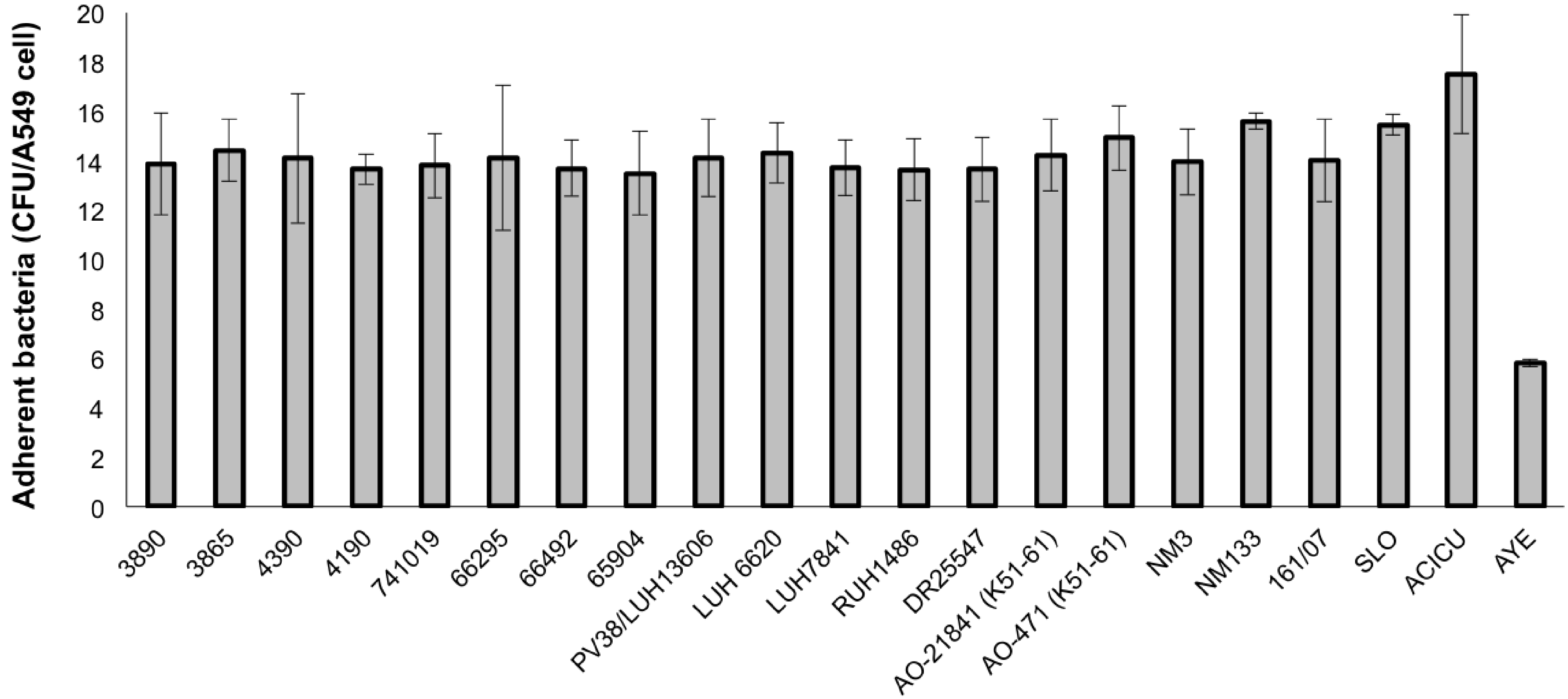

Supplementary Figure S1. Bacterial adherence of *A. baumannii* strains to A549 bronchial epithelial cells. Cell surface-associated bacteri[illegible] after 60 min incubation at 37°C. Error bars represent the standard deviation between triplicates.

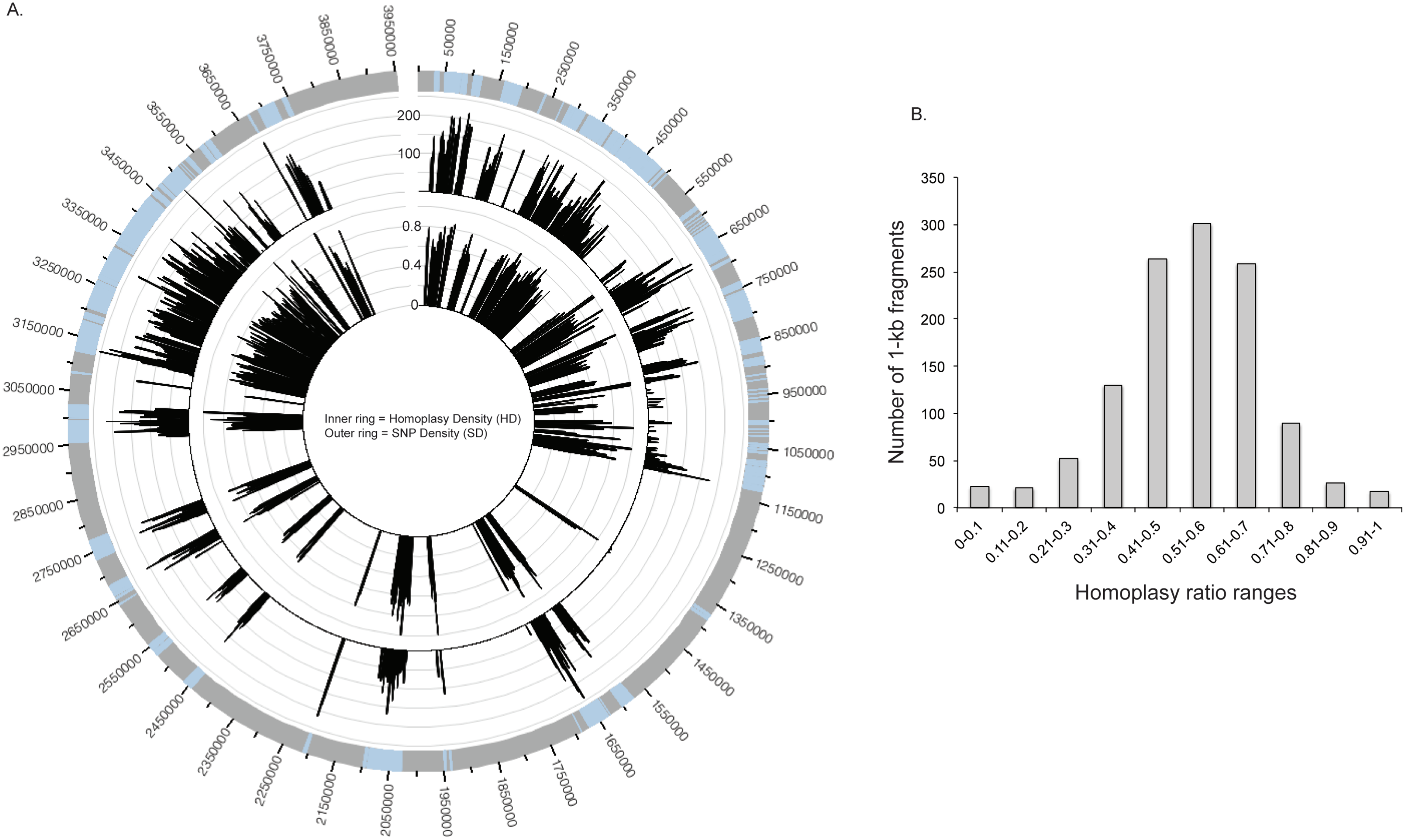

Supplementary Figure S2. Homoplasy density (HD) ratio analysis of queried *A. baumannii* genomes. Panel A: Parsimony informative (PI) single nucleotide polymorphisms (SNPs) were identified across 1-Kb, non-overlapping windows (SNP density, or SD), compared to the reference genome of AB307-0294. Homoplasious SNPs were identified by a Retention index value < 0.05. The HD was calculated by dividing the number of homoplasious SNPs by the total number of PI SNPs. The SD and HD values were visualized with Circos. Core genome regions were identified where there was a call in all query genomes compared to the reference genome. Panel B: The distribution of 1-kb, non-overlapping regions, based on the HD values.

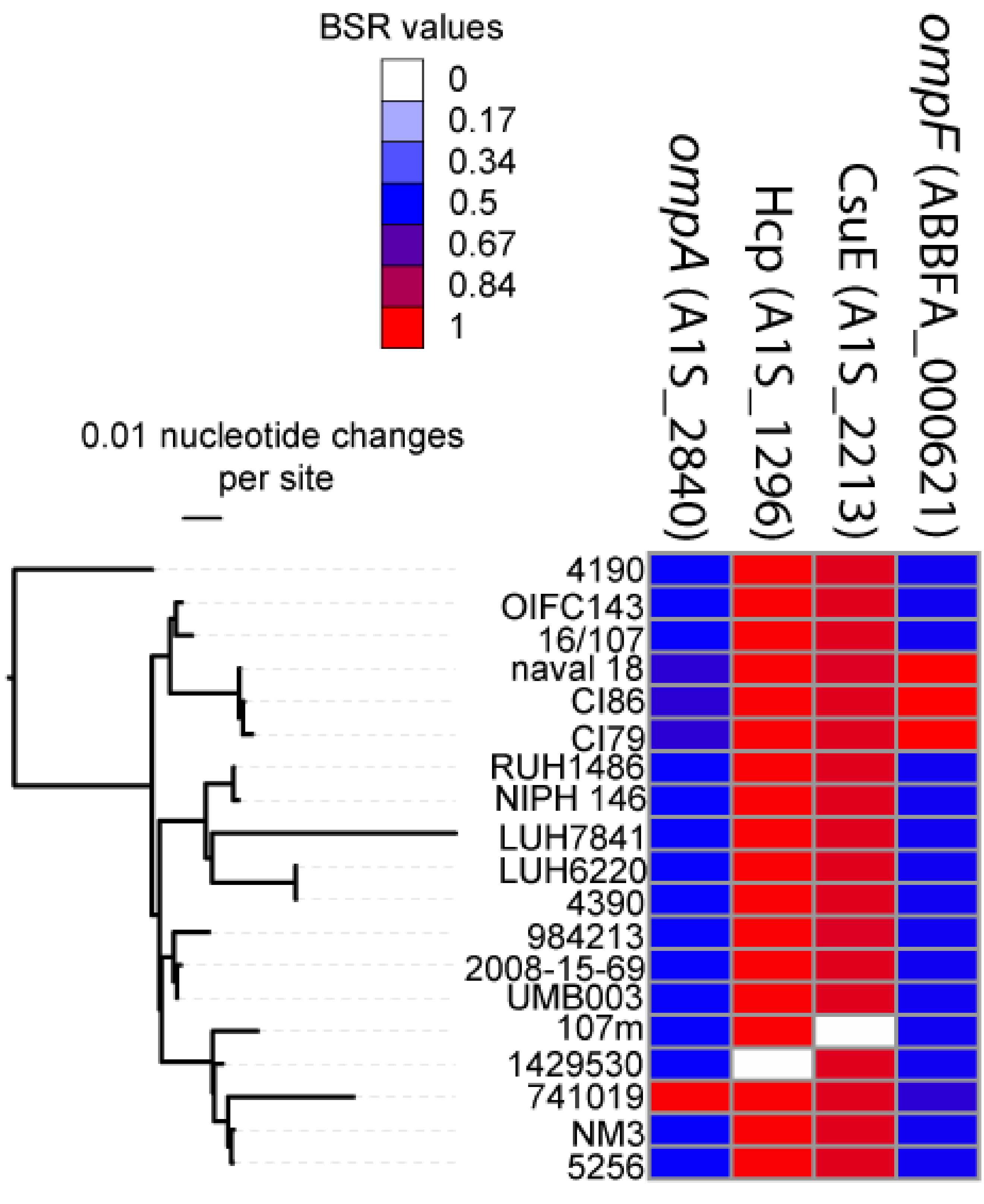

Supplementary Figure S3. The ST25 phylogeny associated with a heatmap of differentially-conserved genes (Supplementary Table S4) associated with virulence. The heatmap was generated from LS-BSR output and was visualized with the interactive tree of life. GenBank accession numbers are listed for each gene.

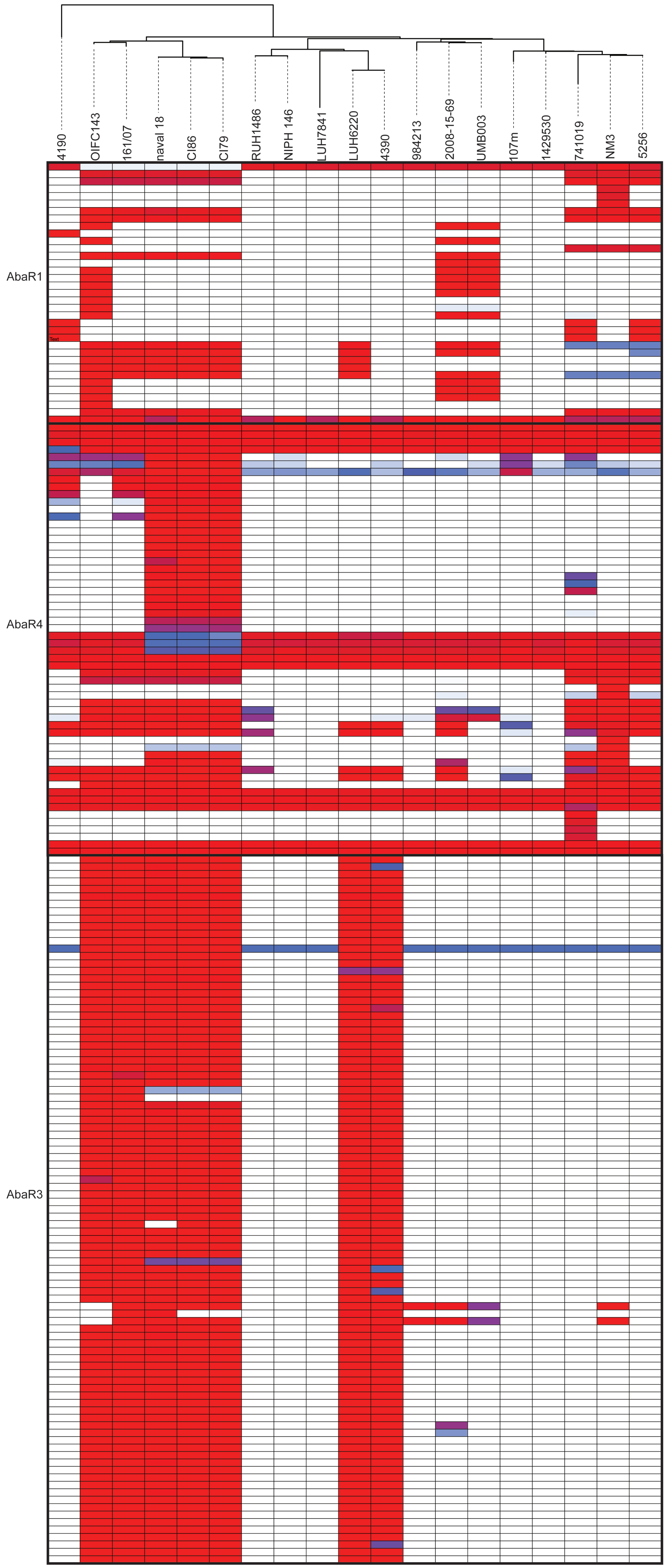

Supplementary Figure S4. The ST25 phylogeny associated with a heatmap of differentially-conserved genes from previously-characterized antibiotic resistance islands. The heatmap was generated from LS-BSR output and was visualized with the interactive tree of life. GenBank accession numbers are listed for each gene.
